# Detection of Parechovirus A1 with Monoclonal Antibody against Capsid Protein VP0

**DOI:** 10.3390/microorganisms8111794

**Published:** 2020-11-16

**Authors:** Ming-Hsiang Kung, Ming-Wei Jan, Jih-Jung Chen, Yi-Chien Shieh, Tsung-Hsien Chang

**Affiliations:** 1 Department of Medical Education and Research, Kaohsiung Veterans General Hospital, Kaohsiung 813, Taiwan; f93626016@ntu.edu.tw (M.-H.K.); davidjan0429@gmail.com (M.-W.J.); 2 Faculty of Pharmacy, School of Pharmaceutical Sciences, National Yang-Ming University, Taipei 112, Taiwan; chenjj@ym.edu.tw; 3 Department of Medical Research, China Medical University Hospital, Taichung 404, Taiwan; 4 Department and Graduate Institute of Microbiology and Immunology, National Defense Medical Center, Taipei 114, Taiwan; w82723@gmail.com

**Keywords:** parechovirus, monoclonal antibody, virus diagnosis

## Abstract

Parechovirus A (PeV-A; human parechovirus) causes mild infections and severe diseases such as neonatal sepsis, encephalitis, and cardiomyopathy in young children. Among the 19 types of PeV-A, PeV-A1 is the most common type of infection. We have previously established an immunofluorescence assay for detecting multiple PeV-A types with a polyclonal antibody against the conserved epitope of VP0. Although the polyclonal antibody is useful for PeV-A diagnosis, it could not distinguish the PeV-A genotypes. Thus, the development of a specific monoclonal antibody for identifying the common infection of PeV-A1 would be beneficial in clinical diagnosis practice. In this study, the recombinant full-length PeV-A1 VP0 protein was used in mouse immunization; a total 10 hybridomas were established. After evaluation by immunoblotting and fluorescence assays, six hybridoma clones with monoclonal antibody (mAb) production were confirmed. These mAbs, which specifically recognize viral protein PeV-A1 VP0 without cross-reactivity to PeV-A3, will prove useful in research and PeV-A1 diagnosis.

## 1. Introduction

Parechovirus A (PeV-A) is a nonenveloped, single-stranded positive-sense RNA virus. It belongs to the *Picornaviridae,* genus *parechovirus*, comprising parechovirus A and B [1]. The PeV-A genome encodes a single polyprotein that is processed into three structural or capsid-encoding proteins (VP0, VP3, and VP1) and seven nonstructural proteins (2A, 2B, 2C (P2); 3A, 3B, 3C and 3D (P3)) [2]. According to the basis of PeV-A genome or viral protein 1 (VP1) sequences, PeV-A1 to PeV-A19 were identified. 

PeV-A1, the most prevalent genotype reported, usually causes asymptomatic or mild gastrointestinal and respiratory diseases, while PeV-A3 triggers more severe consequences, such as central nervous system infection, neonatal sepsis, and even death in infants younger than 3 months [3,4,5]. PeV-A infection threatens the health of young children, and infections of PeV-A in immunocompromised adults have also been reported [6,7]. In addition, hospital-acquired infections in the neonatal department appear to be a critical factor in PeV-A infection [8,9]. Therefore, an effective method for identifying PeV-A infection in hospitals is important for patient care and transmission prevention. 

PCR-based molecular diagnosis is the common approach available for PeV-A detection [10,11]. However, the performance of molecular testing varies according to the virus serotype, subtype, or genotype, especially when diagnosing highly variable RNA viruses such as picornavirus or influenza A virus [12]. Our previous study implemented a comprehensive PeV-A detection approach, involving PCR analysis, virus culture, and immunofluorescence assay, in a clinical virology laboratory. The immunofluorescence assay was conducted with our previously generated polyclonal antibody against the conserved epitope of PeV-A VP0, which recognizes multiple PeV-A serotypes. This polyclonal antibody has been applied in the primary detection of PeV-A in cells [13]. In clinical practices, PeV-A-positive samples identified by immunofluorescence assay require further serotyping by PCR assay, which is a time-consuming procedure that may cause a delay in diagnosis. PeV-A1 is the most prevalent genotype worldwide [5,9,14]; therefore, this study has generated a monoclonal antibody (mAb) that specifically recognizes PeV-A1 but not PeV-A3 for potential clinical applications and further research. This study also reveals that capsid protein VP0 of PeV-A possesses antigenicity, which could be used as a target for antibody-based PeV typing. Thus, to facilitate prompt diagnosis of the PeV-A serotype in clinical practice, a diagnostic monoclonal antibody against a specific serotype of PeV-A VP0 would be useful. 

## 2. Materials and Methods

### 2.1. Virus and Cell Lines

PeV-A1 (strain KVP, GenBank #KC769584.1) and PeV-A3 (strain VGHKS-2007, GenBank #KM986843) were propagated in Vero cells (*Chlorocebus* sp. kidney epithelial cells, ATCC: CCL-81, Manassas, VA, USA) in Dulbecco’s modified Eagle medium (DMEM) supplemented with 10% fetal bovine serum (FBS; Thermo Scientific, Waltham, MA, USA) at 37 °C in a 5% CO_2_ atmosphere [15]. A549 cells (human lung epithelial carcinoma cells, ATCC: CCL-185) were cultured in DMEM supplemented with 10% FBS. 

### 2.2. Generation of Anti-PeV-A1 VP0 Monoclonal Antibody

The full-length cDNA (870 bp) of PeV-A1 VP0 was PCR-cloned and amplified from PeV-A type 1 strain KVP and constructed into the expression vector pET28a (+) after double digestion with restriction enzymes XhoI and NcoI. PeV-A1 VP0 recombinant protein production and purification were conducted using the pET *E. coli* expression system (Novagen, Madison, WI, USA). The purified VP0 protein was immunized in mice to generate hybridomas (Leadgene Biomedical, Inc., Tainan, Taiwan). The studied animals were used in accordance with the protocol approved by the Animal Care and Use Committee of Kaohsiung Veterans General Hospital (#vghks-2019-A011, 6 November 2018). In brief, PeV-A1 VP0 recombinant protein (50 μg) were used for Balb/c mice injection. For antigen priming, the protein was emulsified with Freund’s Complete Adjuvant. After 2 weeks, the antigen was emulsified with Freund’s Incomplete Adjuvant for a booster injection every week. The mice were primed once and boosted 3 times, followed by a bleeding procedure for antibody titration. Then, antiserum samples were harvested to evaluate the antibody specificity and titer. Mice with antibody titers larger than 1:10,000 were further used for hybridoma generation. Splenocytes isolated from the mice were used for generating hybridomas that were fused with hypoxanthine aminopterin thymidine (HAT)-sensitive myeloma cells based on the polyethylene glycol (PEG)-mediated cell fusion approach. Antibodies were screened using antigen-coated ELISA. After several rounds of selection, we used culture supernatants to examine candidates with Western blotting assay. Subsequently, selected candidates underwent expansion for large-scale antibody production and cryopreservation. 

### 2.3. Enzyme-Linked Immunosorbent Assay

PeV-A1 VP0 (5 μg/mL) was coated on the ELISA plate (96 wells) at 4 °C for 16 h. Before measurement, the plate was incubated with a blocking buffer (3% skim milk in phosphate-buffered saline (PBS)) at 37 °C for 1 h. The serially diluted serum (10^−1^~10^−4^) or hybridoma supernatants were added into the wells, followed by incubation at 37 °C for 1 h. After PBS washes, anti-mouse IgG- horseradish peroxidase (HRP, 1:5000, Leadgene, Tainan, Taiwan) was added. After 37 °C incubation for 1 h, the ELISA plate was then washed with PBS. Then, 3, 3′, 5, 5′ tetramethylbenzidine (TMB) subtract was added into the reaction. After incubation at 37 °C for 1 h, the reaction was blocked with a stop solution (1 N H_2_SO_4_). Colorimetric measurement was performed using an ELISA microplate reader at an optical density (OD) of 450 nm (Epoch, Biotek, Winooski, VT, USA). 

### 2.4. Immunofluorescence Assay

Mock- or virus-infected cells were fixed with 4% paraformaldehyde for 30 min, then permeabilized with 0.5% Triton X-100 for 10 min. After two washes with PBS, cells were blocked with 10% skim milk in PBS for 30 min. Cells were incubated with anti-PeV-A VP0 polyclonal antibody (VP0 pAb, 1:500) [15], mouse antiserum, or hybridoma cell supernatant, then Alexa Fluor-568 goat anti-rabbit IgG or anti-mouse IgG secondary antibodies (1:500, Thermo Scientific), each for 1 h in 25 °C. DAPI (1 μg/mL, Sigma-Aldrich, St. Louis, MO, USA) was used for counterstaining. Fluorescence signals were observed and captured by fluorescence microscopy (Axio Observer A1, Zeiss, Oberkochen, Germany).

### 2.5. Immunoblotting Analysis

Cells were lysed in RIPA buffer (150 mM NaCl, 0.5% sodium deoxycholate, 1% NP40, 0.1% SDS, 50 mM Tris-HCl (pH 8.0)) containing a protease inhibitor and a phosphatase inhibitor cocktail (Roche, Basel, Switzerland). A cell extract of 100 μg was separated using 10% SDS-PAGE and transferred to PVDF membranes, which were incubated with anti-PeV-A VP0 polyclonal antibody (VP0 pAb, 1:500), mouse antiserum, or hybridoma cell supernatant, then HRP-conjugated goat anti-rabbit or mouse IgG secondary antibody (Jackson ImmunoResearch Laboratory, West Grove, PA, USA), and visualized using an enhanced chemiluminescence system (Advansta, San Jose, CA, USA). Images were acquired using a digital image system (UVP, LLC).

## 3. Results

### 3.1. Generation of PeV-A VP0 Hybridoma

**Figure 1:**
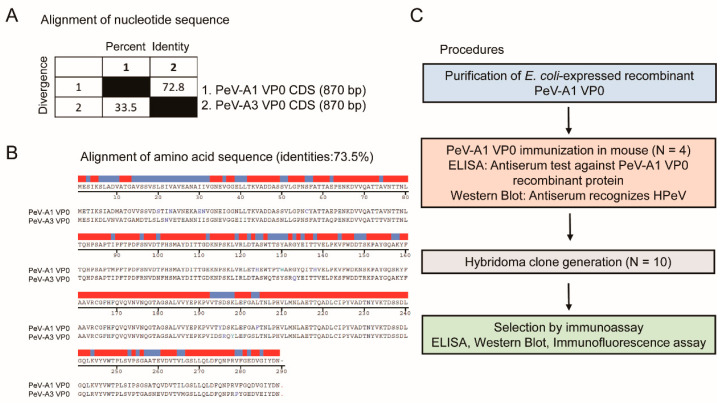
Generation procedures of PeV-A1 VP0 hybridoma. (**A**,**B**) Nucleotide and amino acid sequence similarity of VP0 from PeV-A1 and PeV-A3. Red and blue bands indicate identical and nonidentical amino acid residues, respectively. (**C**) Schematic for hybridoma generation.

The VP0 polypeptide of PeV-A possesses antigenic properties [13,16]. Comparison of the VP0 nucleotide sequence of PeV-A type 1 (strain KVP, GenBank #KC769584.1) and type 3 (strain VGHKS-2007, GenBank #KM986843) showed 72.8% identity and 33.5% divergence (Figure 1A). The alignment analysis of the amino acid sequence indicated 73.5% homology between PeV-A1 and PeV-A3 VP0 (Figure 1B). These findings suggested potential feasibility in generating a specific mAb against PeV-A1 VP0. The workflow of the generation of the PeV-A1 VP0 mAb is summarized in Figure 1C. The whole VP0 recombinant protein was employed to elicit antibody production in mice (*N* = 4). Antiserum was utilized to analyze its targeting efficacy and specificity against PeV-A1 VP0 using ELISA and Western blot. Splenocytes from the VP0-immunized mouse were isolated for hybridoma generation. Ten hybridoma clones were obtained, and mAb produced by them were harvested for further evaluation. 

### 3.2. Evaluation of PeV-A1 VP0 Antiserum 

**Figure 2:**
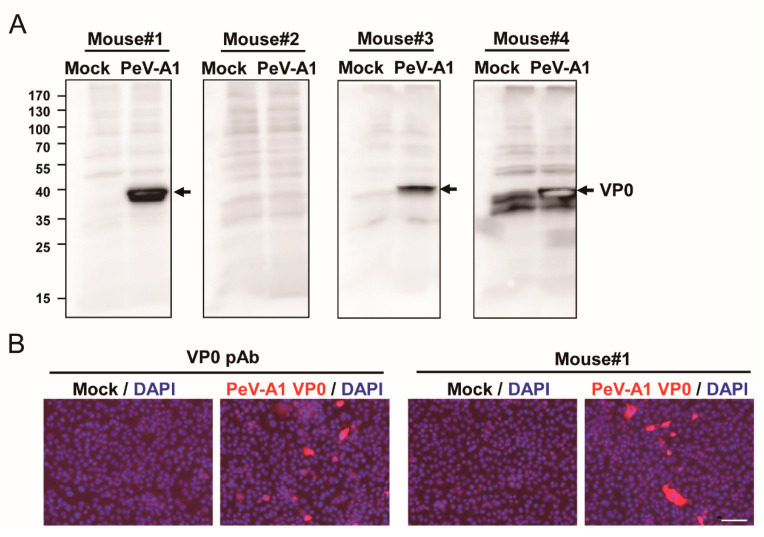
Evaluation of mouse antiserum against PeV-A1 VP0. (**A**) A549 cells (1 × 10^5^) were infected with mock or PeV-A1 (MOI = 5). Cell lysates were harvested at postinfection 24 h for VP0 detection by antiserum (dilution factor of 1:5000) from four immunized mice. Arrow: PeV-A VP1. (**B**) Immunofluorescence assay was conducted in PeV-A1-infected A549 cells (1 × 10^5^) for 24 h. Both PeV-A VP0 polyclonal antibody (left panels) and Mouse #1 antiserum (right panels) were used (dilution factor of 1:500). The fluorescence-merged images of PeV-A1 VP0 (red) and DAPI (nuclear, blue) are shown. Scale bar, 100 μm.

To evaluate the production of anti-PeV-A1 VP0 antibody in the immunized mice, the antiserum (dilution 1: 5000) from four mice were employed to detect the VP0 in PeV-A1-infected cell lysates. Immunoblotting assay results showed that except for Mouse #2, antiserum of immunized Mouse #1, Mouse #3, and Mouse #4 detected PeV-A1 VP0 in the proper molecular weight (~32 KDa; Figure 2A). However, the nonspecific reactivity in mock infection was seen in the antiserum of Mouse #4. Similar results were obtained using ELISA assay; the antiserum (1 × 10^4^ dilution) of Mouse #1, Mouse #3, and Mouse #4 reacted to VP0 recombinant proteins. Antiserum of Mouse #2 showed a below-average, lowest absorbance value in wavelength OD 450 nm (Figure S1). Immunoblot and ELISA data indicated that VP0 immunization induced a limited level of antibody production in Mouse #2. However, the 75% immunization rate revealed high antigenicity of PeV-A1 VP0. Comparison of antigen-recognition patterns in the antiserum of Mouse #1, Mouse #2, and Mouse #4 showed a higher detection signal of VP0 and a lower nonspecific reaction in Mouse #1. Therefore, Mouse #1 antiserum was further subjected to immunofluorescence assay; the polyclonal antibody against PeV-A VP0 was used as a positive control. The images obtained showed PeV-A1-infected cells detected by Mouse #1 antiserum. The polyclonal PeV-A VP0 antibody was used as a positive control (Figure 2B). 

### 3.3. Validation of PeV-A1 VP0 Hybridoma 

**Figure 3:**
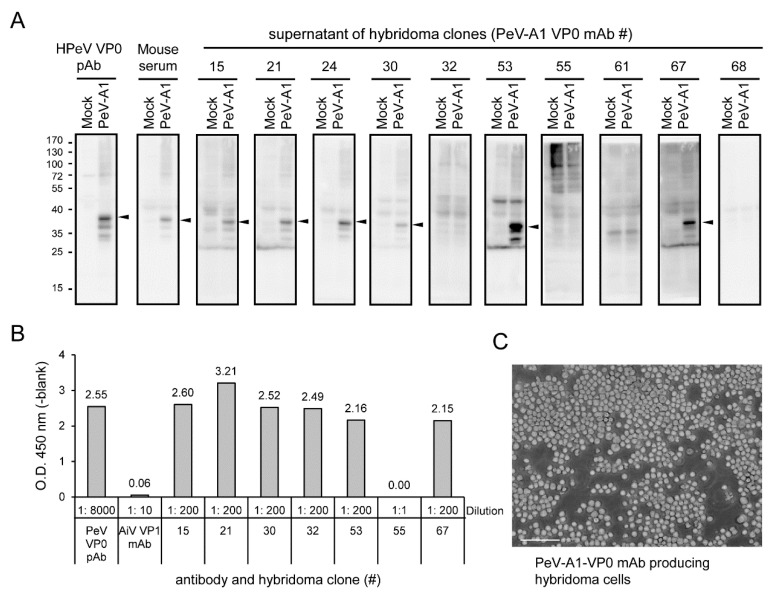
Identification of anti-PeV-A1 VP0 monoclonal antibody (mAb)-producing hybridoma clones. (**A**) A549 cells were infected with mock and PeV-A1 (MOI = 5) for 24 h; cell lysates were separated in SDS-PAGE for Western blot with hybridoma mAb supernatant, polyclonal VP0 antibody, or mouse antiserum. The arrow indicates the detected PeV-A1 VP0 protein. (**B**) ELISA assay was conducted with hybridoma mAb supernatant, polyclonal PeV-A VP0 antibody, or Aichi-VP1 mAb. The dilution factor of each antibody is indicated. The absorbance value of O.D.450 (-blank) is shown. (**C**) The representative image of hybridoma growth is shown. Scale bar, 100 μm.

According to antiserum evaluation results, Mouse#1 was selected for hybridoma generation. Ten hybridoma clones were generated after the entire process. The culture supernatants of these hybridomas were harvested to evaluate the anti-PeV-A1 VP0 mAb production. Immunoblotting showed that 6 of the 10 clones (Clones #15, #21, #24, #30, #53, and #67) were able to detect the VP0 in PeV-A1-infected cells (Figure 3A). These 6 clones of PeV-A1 VP0 mAb were further confirmed by ELISA assay. In the tests, Clone #55 could not detect PeV-A VP0 in immunoblotting or ELISA. The polyclonal PeV-A VP0 antibody was used as the positive control [13], and Aichi virus VP1 mAb was used as the negative control (Figure 3B). The present findings suggest that PeV-A1 VP0 mAb can be used in Western blot and ELISA assays. The image of hybridoma morphology showed cells in a well-grown culture condition (Figure 3C). 

### 3.4. Specificity of PeV-A1 VP0 mAb

**Figure 4:**
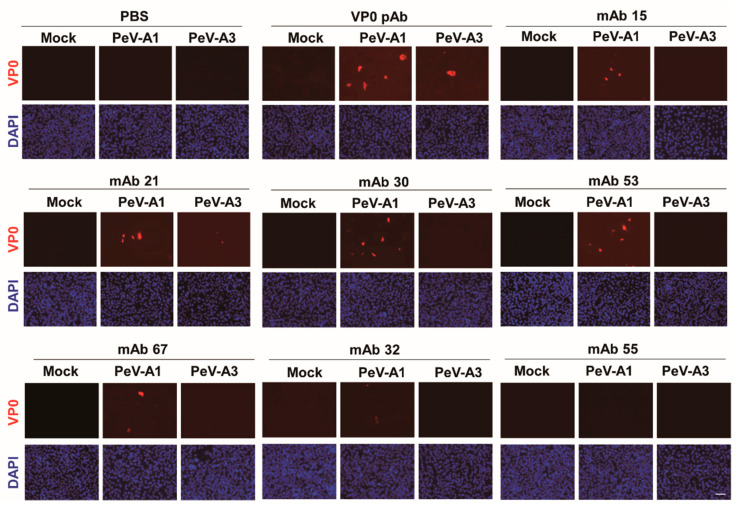
Detection of PeV-A1 by anti-PeV-A1 VP0 mAb. Mock-, PeV-A1-, and PeV-A3-infected A549 cells, after 24 h, were subjected to immunofluorescence analysis with hybridoma mAb supernatant (dilution factor = 1:10) and polyclonal VP0 antibody (dilution factor = 1:500). The polyclonal VP0 antibody detected PeV-A1 and PeV-A3 (red). PeV-A1 VP0 mAb detected PeV-A1- but not PeV-A3-infected cells. DAPI showed nuclear staining (blue). Scale bar, 100 μm.

**Figure 5:**
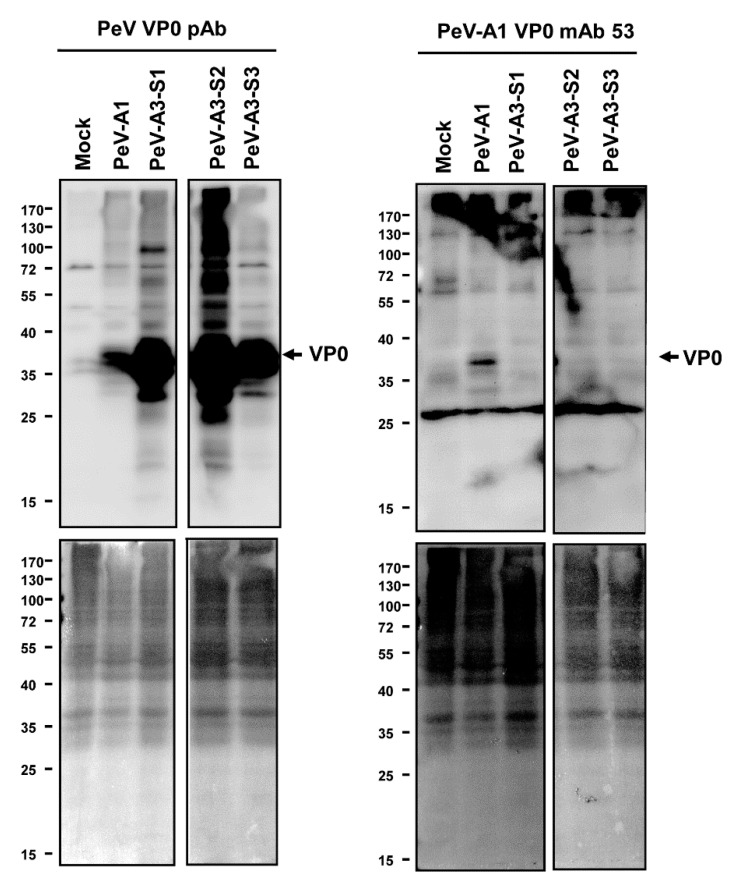
Anti-PeV-A1 VP0 mAb detects PeV-A1 but not PeV-A3. Immunoblotting assay was conducted in mock-, PeV-A1-, and PeV-A3-infected A549 cells for 24 h. Both hybridoma mAb supernatant and polyclonal VP0 antibody were employed to recognize PeV-A VP0 (**upper panel**). The protein-loading control (Ponceau S. staining) is shown in the **lower panels**.

Immunofluorescence assay was performed to verify the PeV-A1 recognition specificity of these mAb clones. Results showed that Clones #15, #21, #24, #30, #63, and #67 were able to detect PeV-A1-infected cells but not PeV-A3-infected ones. Negative Clones #30 and #32 showed no positive-staining (Figure 4). Moreover, immunoblot analysis also demonstrated the specificity of PeV-A1 VP0 mAb. Only PeV-A1 VP0 was recognized but not the other three isolated strains of PeV-A3, which were detected by the PeV-A VP0 polyclonal antibody (Figure 5). These results revealed that the newly generated PeV-A VP0 mAb showed no cross-reactivity to PeV-A3 and, hence, can be used as the specific antibody in the detection of PeV-A1.

## 4. Discussion

PeV-A1 is the main cause of PeV-A disease in children under the age of 2 years [5]. Developing a specific detection tool for diagnosing PeV-A1 infection is of need and importance. In this study, anti-PeV-A1 mAb-producing hybridomas were generated using PeV-A1 VP0 recombinant protein. A total of six hybridoma clones were developed. These mAb-specific clones detected PeV-A1 VP0 without cross-reactivity to PeV-A3 and can be used in applications of PeV-A1 ELISA, immunoblotting, and immunofluorescence assays. Upon further evaluation, the new mAb clones would be useful for PeV-A1 detection in clinical laboratories. 

A comprehensive PeV-A diagnosis protocol has been established in a clinical virology laboratory, involving viral culture, cytopathic effect, immunofluorescence assay, PCR analysis, and genotype sequencing. Among these approaches, the PeV-A polyclonal antibody was applied in an immunofluorescence assay. This PeV-A polyclonal antibody recognizes the conserved epitope existing in the capsid protein VP0 of multiple PeV-A serotypes. The diagnosis protocol was used in our routine clinical practice of PeV-A and enterovirus identification, facilitating the process of PeV-A infection diagnosis [13]. However, this polyclonal antibody is not able to identify the different PeV types, thus necessitating a PCR analysis. The PeV-A1 VP0 mAbs developed in this study contribute to a more efficient PeV-A1 diagnosis. The monoclonal antibody against PeV-A3 VP0 has been previously reported by Goto K. et al. [17]. They used cell-free, wheat-germ-synthesized PeV-A3 VP0, with antigenicity for immunizing BALB/c mice to generate hybridomas. The produced PeV-A3 VP0 mAb was evaluated by an ELISA assay, which showed no cross-reactivity to other related viruses [17]. Informatively, the PeV-A3 VP0 mAb-recognized epitopes were mapped. Their study demonstrated that the PeV-A3 VP0 mAb is a useful tool for PeV-A3 detection; we thought that it would be critical in the clinical diagnosis of PeV-A3 infection. However, whether the PeV-A3 VP0 mAb was used in the immunoblotting and immunofluorescence assays remains unclear.

Two mAbs were found to have a neutralization effect against PeV-A1 and PeV-A2. One recognized a linear epitope of PeV-A1 VP1, and the other identified the PeV-A2 quaternary epitope on the VP0 and VP3 loops [18]. The virus neutralization effects of the PeV-A1 VP0 mAb developed in this study and the reported PeV-A3 mAb require further investigation. Moreover, the current results reveal that PeV-A1 VP0 mAb showed no reactivity to PeV-A3. 

As the PeV-A1 VP0 mAb only tested PeV-A1 and PeV-A3 to evaluate its specificity, the limitation of this study is whether PeV-A1 VP0 mAb cross-reacts with other picornaviruses, which needs to be further evaluated. In addition, the PeV-A1 VP0 mAb-recognized epitopes need to be identified. 

In conclusion, the successful development of PeV-A1 mAbs in this study contributes to prompt PeV-A diagnosis and advances in research. We also revealed that capsid protein VP0 could be used as an antibody-targeting protein to distinguish different serotypes of PeV-A. 

## Supplementary Materials

The following are available online at https://www.mdpi.com/2076-2607/8/11/1794/s1. Figure S1: ELISA assay using mouse antiserum.
