# Supplementary material for: Detection of Parechovirus A1 with Monoclonal Antibody against Capsid Protein VP0
Source: Microorganisms. 2020 Nov 16;8(11):1794. doi: 10.3390/microorganisms8111794 (PMC7696872; doi:10.3390/microorganisms8111794)
Supplement: Supplementary file 1 [file microorganisms-08-01794-s001.pdf]

Supplementary information

Figure S1

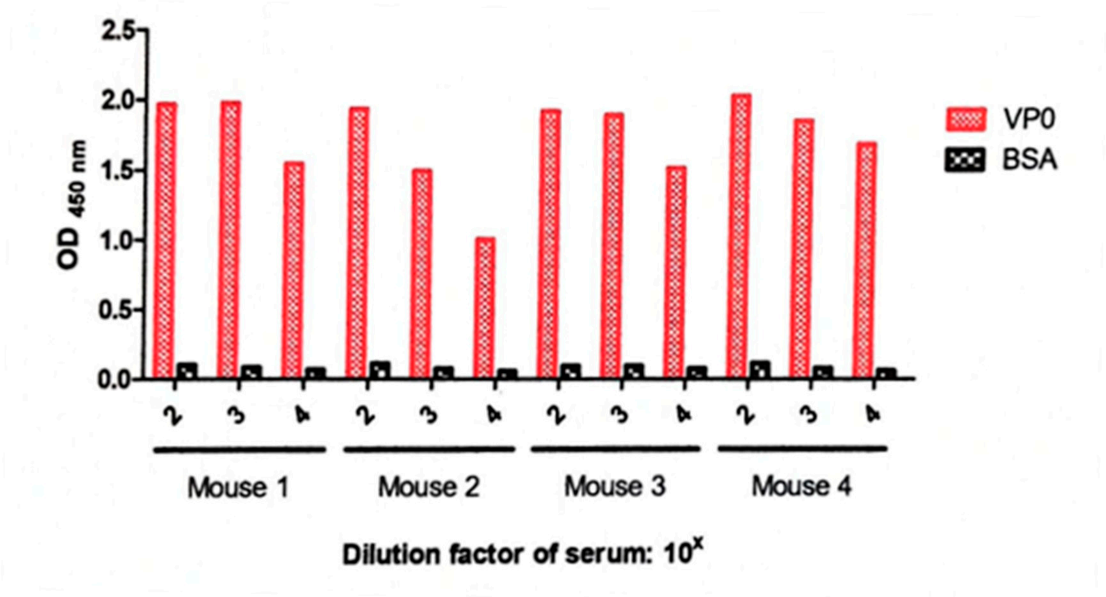

Figure S1. ELISA assay using mouse antiserum.

The mouse antiserum (10^{-2}~10^{-4} dilution) were added into the PeV-A1 VP0 antigen and BSA (negative control)-coated ELISA plate. After reaction, the absorbance value of O.D.450 was measured.
